# Laparoscopic Ureteral Reimplantation in Endometriosis: A Severe Case

**DOI:** 10.1055/s-0037-1607296

**Published:** 2017-11-27

**Authors:** Garri Tchartchian, Paul G. Fabricius, Bernd Bojahr, Rudy L. De Wilde

**Affiliations:** 1 Klinik für Minimal Invasive Chirurgie, Berlin-Zehlendorf, Germany; 2 Klinik für Frauenheilkunde und Brustzentrum, Universitätsklinikum Greifswald, Greifswald, Germany; 3 Klinik für Frauenheilkunde, Geburtshilfe und Gynäkologische Onkologie, Universitätsklinik für Gynäkologie, Pius-Hospital, Oldenburg, Germany

**Keywords:** endometriosis, laparoscopy, ureteral reimplantation

## Abstract

In aggressive cases, endometriosis can perturb the urogenital tract, in particular the ureter, which can potentially result in ureteral compression or stenosis. Even though this is rare, consequences are dramatic, such as hydronephrosis or organ failure. The present standard intervention comprises the resection of affected tissues and endometriosis foci combined with adjuvant hormonal therapy. When the ureter does not recover, ureteral reimplantation is required. The present case describes the successful laparoscopic approach of the reimplantation of the ureter with simultaneous cystoscopy.

Endometriosis refers to all diseases where extrauterine growth of uterine mucosa results in painful, chronic inflammation. 1 Even though endometriosis foci are benign, these are characterized by an aggressive and infiltrating growth. These mainly spread in the pelvic organs and the peritoneum, yet rarely, endometriosis can affect other areas of the body outside of the pelvic region as well. 2 In less than 0.5% of all cases, endometriosis perturbs the urogenital tract, in particular the ureter; yet, the bladder and the kidneys can be affected as well. When this results in ureteral compression or stenosis, it inevitably leads to hydronephrosis with risk of organ failure. 3 Although this complication is rare, it remains tragic, even more so because it can be avoided by consistent monitoring. Nowadays, because of accumulation of patients and the relatively large number of cases in endometriosis clinics, urological complications are no longer a rare occurrence.

The present therapeutic recommendations comprise laparoscopy as the central diagnostic element, combined with the surgical removal of the affected tissues. 4 In case the bladder is affected as well, resection of the bladder wall is performed in most cases, and when the corresponding kidney still functions sufficiently, a ureteral stent is placed. When the ureter does not recover and the adjuvant hormone therapy does not have the desired effect, a ureteral reimplantation should be considered. In that case, the affected segment of the ureter is excised and the remaining healthy part is anastomosed with the bladder. The present severe endometriosis case describes a successful laparoscopic approach of ureteral reimplantation.

## Case Report

During our regular interdisciplinary endometriosis discussion rounds, we were presented with a 39-year-old patient who suffered from chronic pain, which she rated as 7 to 8 on the visual analog scale (VAS) of 0 to 10 (0 correlating with no pain and 10 severe unbearable pain). Her previous medical history included a laparoscopic endometriosis clearance, with resection of the visible foci, performed in 2001. These included foci in both fossa ovarica, pouch of Douglas, plica vesicouterina, and left pelvic wall, which were removed by means of bipolar coagulation. Endometriotic foci in the vicinity of the left ureter, however, were only treated superficially. Postoperatively, open surgery, which would allow for exposure of the ureter, was suggested, yet firmly refused by the patient. Instead, to relieve the left hydronephrosis (observed as grade I–II), double J stents were placed and administration of gonadotropin-releasing hormone (GnRH) analogues was initiated. Medical therapy was continued for 6 months, after which therapy was extended with the combined oral anticontraceptive pill.

Noteworthy comorbidities are Hashimoto's thyroiditis and arterial hypertonia. In 2005, given the existing urological problems and a suspected nephrolithiasis, an extracorporeal shockwave lithotripsy (ESWL) was performed in the left kidney after a new ureteral stent was placed. According to the patient, however, no kidney stones or stone fragments were removed during that treatment. After the placement of the double J stents, a renal scintigraphy showed that the flow rate of left kidney was 40%; after furosemide administration, the renal flow rate was adequate. Nevertheless, as before, the organ was still dilated. The patient denied further surgical interventions, although the kidney and ureter functions were continuously monitored and did not reveal any significant regression.

**Fig. 1:**
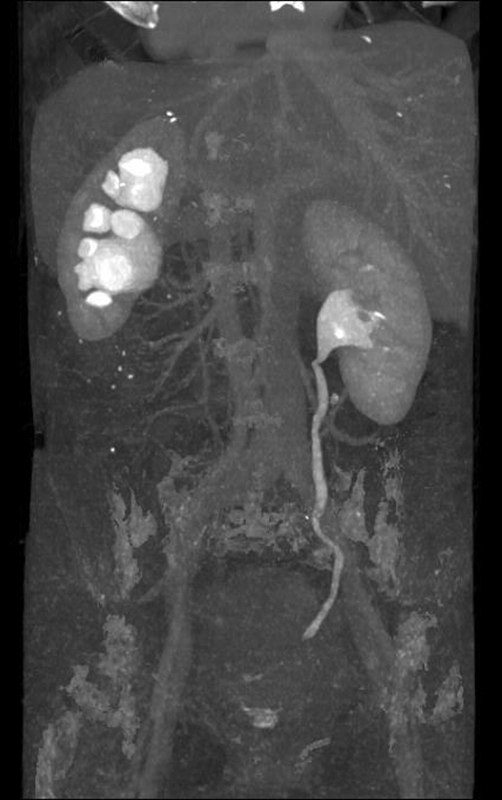
CT, September 2014. CT, computed tomography.

**Fig. 2:**
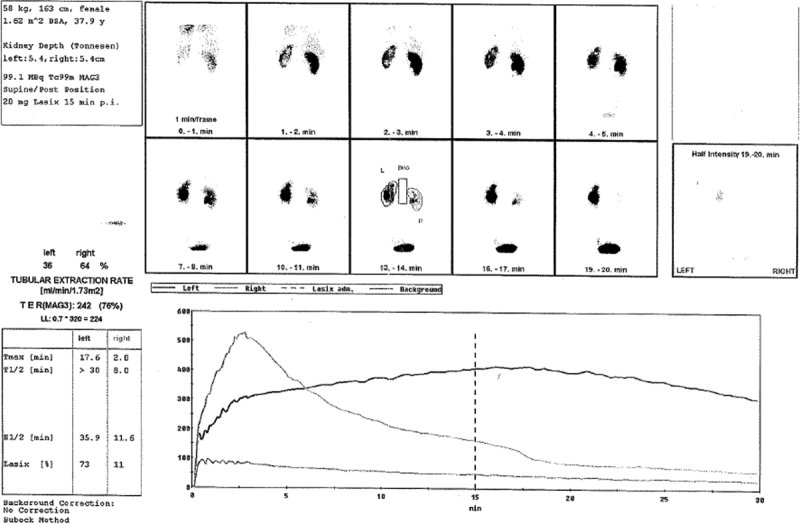
Renal scintography shows a flow rate of merely 36% in the left kidney.

However, increased complaints in 2014 demanded a visit to our clinic. We suspected an advanced endometriosis coinciding with rectovaginal nodule and ureteral and periureteral infiltration resulting in the formation of hydroureter as well as hydronephrosis grade II as shown by computed tomography (CT, Fig. 1 ). Scintigraphy of the left kidney showed a flow rate of merely 36% ( Fig. 2 ). Furthermore, her blood creatinine level was elevated to 1.05 µg/dL.

We chose to perform a laparoscopic procedure to remove all visible and palpable endometrioma inside and outside the pelvis as well as ureterolysis and intraoperative resection of the rectovaginal nodule. On top, supracervical hysterectomy, because of suspected adenomyosis, was performed. Simultaneously, we placed a 6-Fr double J stent in the left ureter to relieve the left kidney. The latter procedure was strenuous and could only be done after the corresponding endometriomas were laparoscopically removed. There were no postoperative complications, and the patient experienced a dramatic improvement of all symptoms, including a significant decrease in pain (VAS 2). After 6 months, the double J stent was removed. Initially, the patient remained free of symptoms and no deterioration of hydronephrosis was observed.

Nevertheless, 1 year later, a kidney scintigraphy showed a further deterioration of the kidney flow rate to 27% (ÓReilly grade II) and an increasing obstruction.

**Fig. 3:**
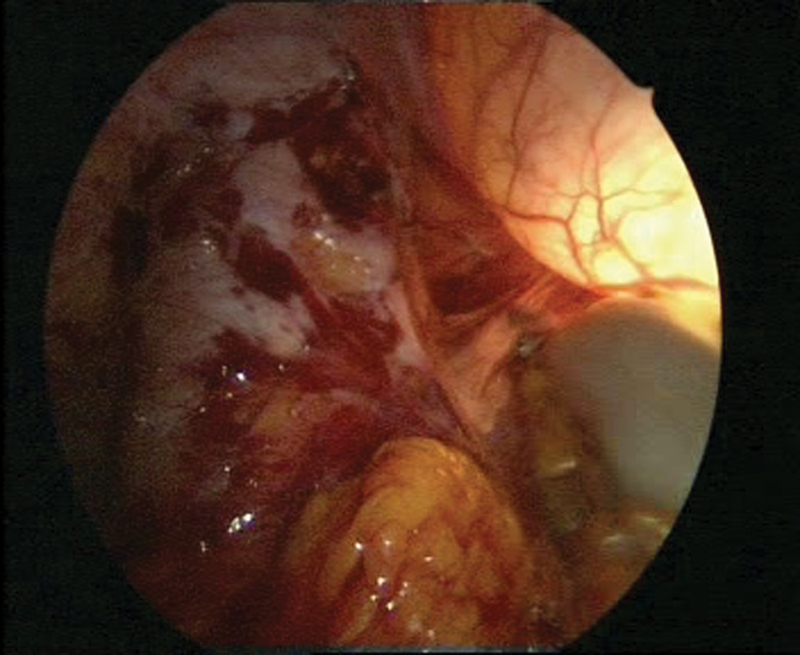
Laparoscopic surgery with simultaneous cystoscopy.

Despite furosemide treatment, hardly any flow was observed in the left kidney. As such, the prevesical ureter was not only restenosed, but also further reduced in its function because of limited peristalsis. To address this, we suggested a laparoscopic ureteral reimplantation surgery ( Figs. 3 4 5 6 7 8 9 10 11 12 13 14 ). At this point, it is important to mention that we had suspected the ureter infiltration to have been present for several years already. Yet, for over more than a decade, the patient firmly refused any form of surgical intervention, even though multiple clinicians and centers recommended surgery and warned her about potential risks and consequences of not doing so. Resection of the affected ureter, when infiltration was suspected and obstructive uropathy or even hydronephrosis occurred, should have been performed immediately. 5

**Fig. 4:**
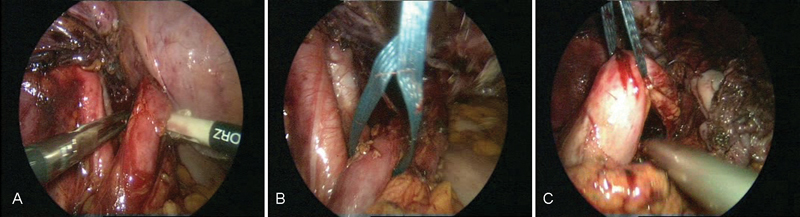
( **A** – **C** ) Exposure of the left ureter.

**Fig. 5:**
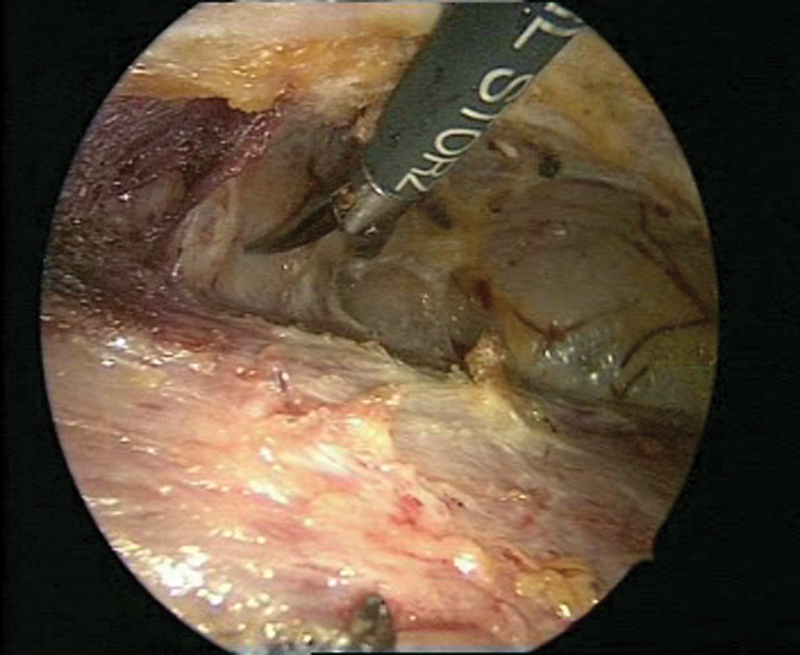
Preparation of the bladder for the psoas hitch procedure.

**Fig. 6:**
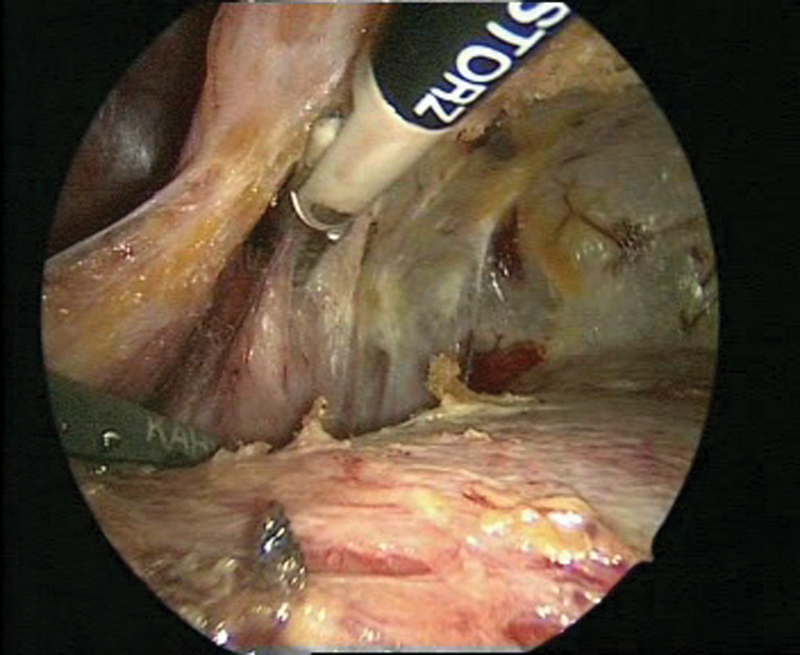
Preparation of the Retzius space and exposure of the bladder.

**Fig. 7:**
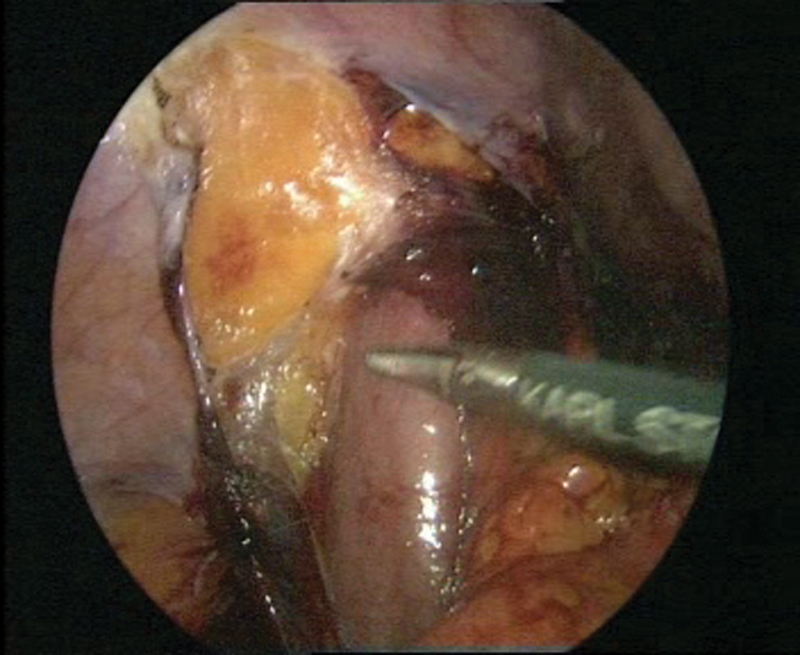
Preparation of the left psoas muscle.

**Fig. 8:**
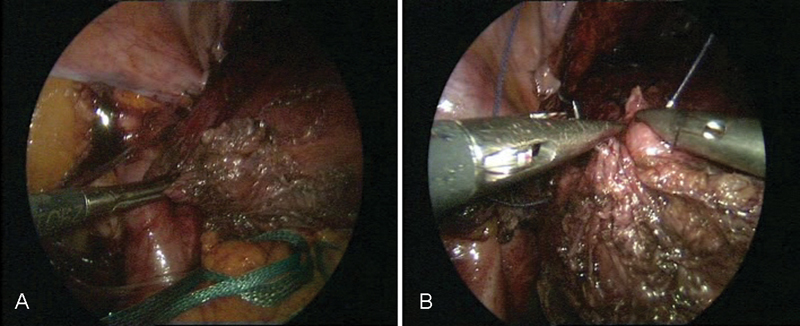
( **A** – **B** ) Fixation of the prepared bladder to the left psoas muscle.

**Fig. 9:**
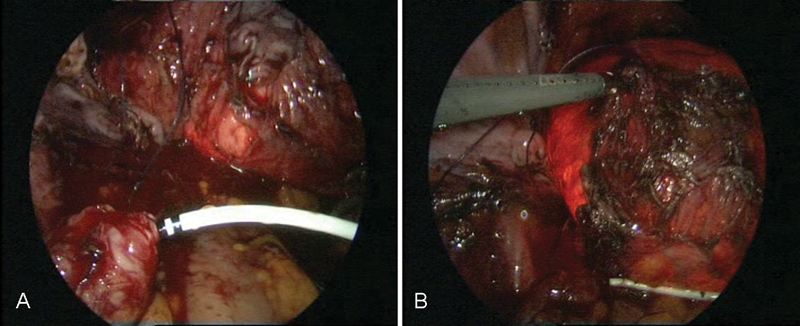
( **A** – **B** ) Reimplantation of the left ureter with simultaneous cystoscopy.

**Fig. 10:**
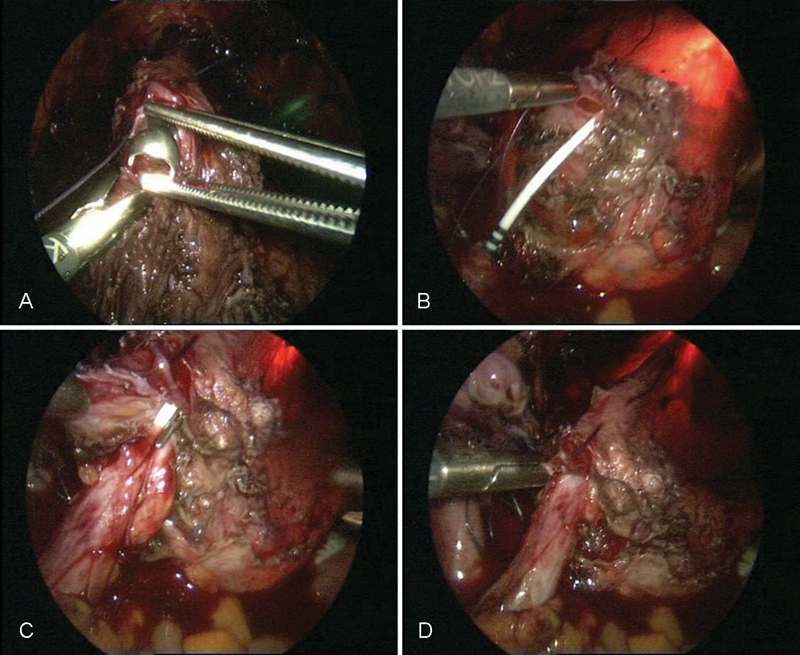
( **A** – **D** ) Cystoscopy during laparoscopic ureteral reimplantation of the ureter.

**Fig. 11:**
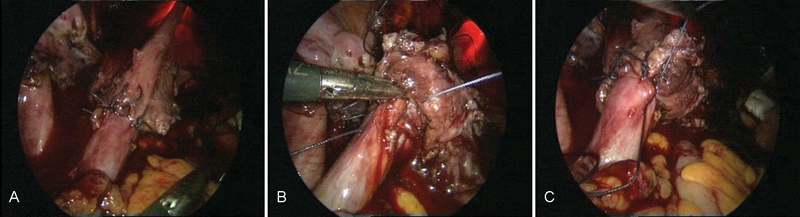
( **A** – **C** ) Anastomosis in the vicinity of the left ureteral reimplantation.

**Fig. 12:**
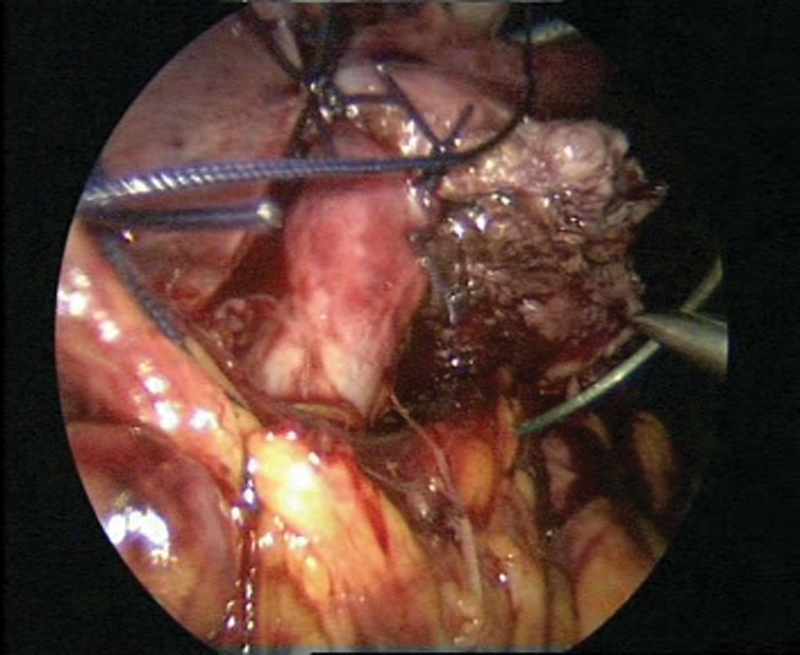
Fixation to the psoas muscle.

**Fig. 13:**
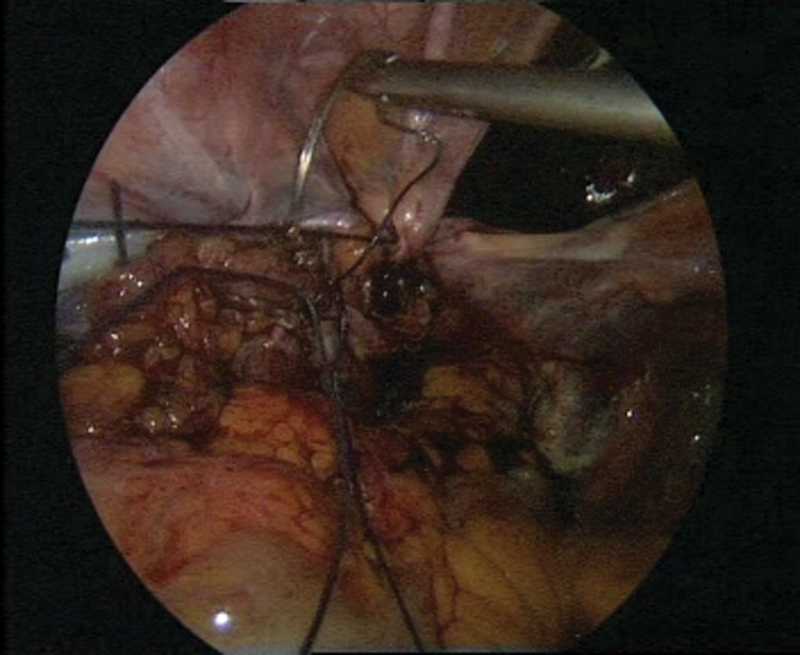
Closure of the peritoneum while keeping the anastomosis extraperitoneally.

**Fig. 14:**
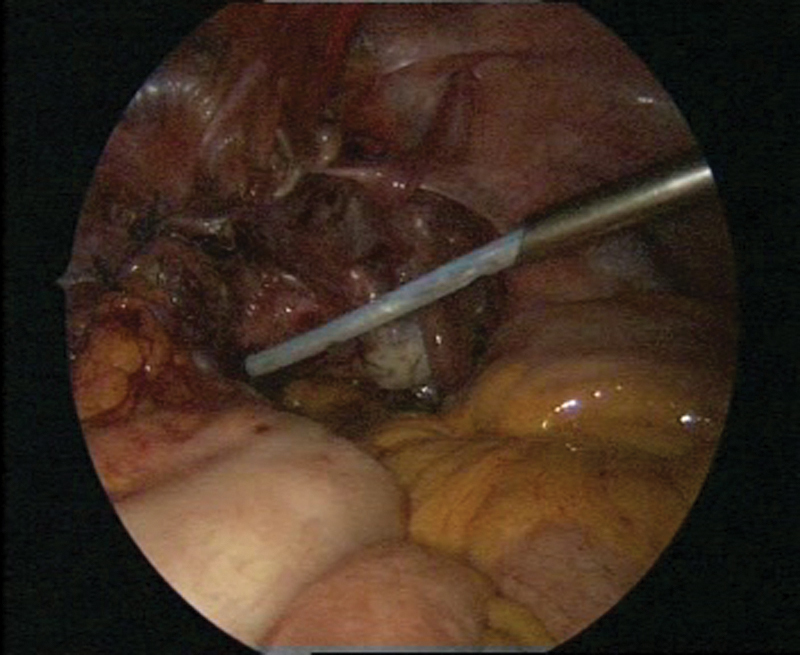
Final stage of the procedure.

The procedure was performed as follows. After cystoscopy and placement of yet another 6-Fr double J stent, the endometriomas in the pelvis were removed during laparoscopic exploration. The juxtavesical ureter was fixed in endometriosis tissue. Only at the level of the vessel intersection, we found sufficiently large inconspicuous anatomic conditions of the ureter, which could serve for reimplantation. Thus, to reach the bladder, a distance of at least 10 cm had to be bypassed. In such cases, the psoas hitch procedure is indicated. 6 We chose to perform this technique laparoscopically as opposed to the usual open surgical approach. For this purpose, the entire bladder was mobilized toward the lateral right wall, to be pulled toward the likewise exposed psoas muscle (“Hitch” preparation). Next, the hitch suture fixation was applied on the left bladder wall, so it could be attached to the psoas muscle at the end of the operation. This was an important step, as this part of the bladder was hard to reach after completion of anastomosis. While sparing the double J stent, the already mobilized ureter was severed shortly below the vessel intersection, and the stent was carefully pulled from the bladder. After closure of the distal ureter with a Roeder knot, the bladder was opened below the fixation sutures with endoscopic assistance, keeping the perforation small. As such, we gained access to the ureter lumen, and by use of forceps, the double J stent-equipped ureter could be pulled from the abdominal cavity into the bladder. The ureterocystostomy was performed according to Dreikorn et al; 7 the ureter was merely fixed extravesically and then latched onto the bladder without tunneling. The anastomosis was done with a total of eight 3–0 sutures. First, two anterior sutures were made to compose the entire bladder and fix the ureter in such a manner that it could be integrated into the bladder wall. Then, each suture was repeated laterally, medially, and posteriorly. After testing the integrity of the sutures with cystoscopy, the psoas bladder hitch fixation suture was fixed and attached to the psoas. Because of applying the psoas hitch procedure, the anastomosis was free of tension. Next, the placement of an extraperitoneal Robinson drainage and the closure of the peritoneum were performed so that the anastomosis and the drainage were lying extraperitoneally.

**Fig. 15:**
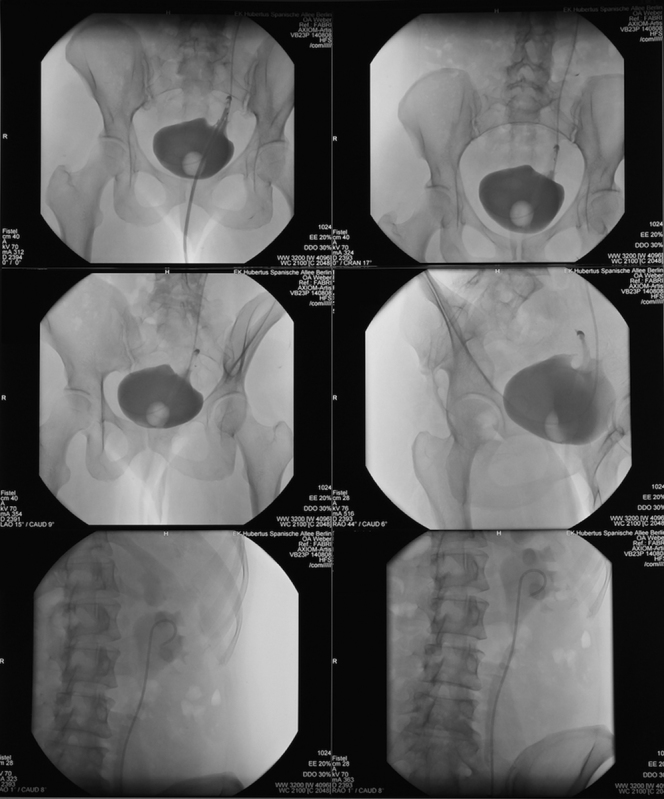
Cystography after ureteral reimplantation.

No postoperative complications occurred. After cystography ( Fig. 15 ) and contrast CT of the urinary tract, the patient could be discharged after 8 days of hospitalization and transferred to outpatient care without any discomforting symptoms.

The patient continued to be monitored during follow-up. Her creatinine levels returned within the normal range. Examinations of the renal flow showed no aberrations; yet, the hydronephrosis persisted as a “morphologically fixed” aberration due to 15 years of dilatation, and the function of the left kidney remained reduced at 31%. The hormone therapy was no longer continued.

## Discussion

It is estimated that 0.3 to 12% of women diagnosed with endometriosis also have urinary tract involvement. This number increases to 14 to 20% in patients with deep infiltrating endometriosis. 8 After the bladder, the ureter is the second most common affected site. Ureter infiltration is often asymptomatic; yet, it is key to diagnose and treat promptly to avoid complications, such as stenosis with hydroureter, hydronephrosis, and eventually loss of renal function. 9 Because complaints not always point in the direction of problems of the urinary tract, it is imperative to conduct regular sonographic monitoring in patients with endometriosis, including in their urinary tract. At our clinic, as in other endometriosis centers, these examinations became routine. In the present case, the patient firmly refused any kind of surgical intervention for over a decade after initial laparoscopic endometriotic foci clearance, which indicated potential ureter involvement.

Nowadays, the standard treatment of endometriosis comprises complete surgical removal of endometriosis tissue followed by conservative hormone therapy. Regarding the surgical intervention, preference is given to minimally invasive techniques. 10 11 12 13 As such, open surgery approaches have been almost completely abandoned in our clinic, which is in line with recommendations of other clinics. The aim of the surgical treatment is an optimal surgical and therapeutic outcome, which results in an increase of quality of life with an efficient pain treatment policy of the disease. 4 14

Invasion of the urinary tract should always be ruled out, even when the case history does not suggest any abnormalities. In case of endometriosis-related changes in the urogenital system, the primary concern is always the preservation and conservation of the renal function. 15 Disorders of the upper urinary tract are almost exclusively caused by ureteral infiltration in the pelvic region. Initially, ureteral stents are justified, especially while waiting for successful results of surgical removal of endometriomas and adjuvant hormone therapy. However, when placement of the double J stent does not result in sufficient renal flow, ureteral stents present no long-term solution in the relatively young patients. When the kidney's urinary retention increases or tubular function deteriorates, an adequate surgical intervention is required. 16 When renal scintigraphy reveals that the conservation of the organ is justified (>25% of its normal function), the ureter should be cleared in such a manner so that flow does not reduce any further. Because the problem is situated in the pelvic ureter, the primary therapeutic choice is ureteral reimplantation. A ureteral resection with end-to-end anastomosis could be unsuccessful owing to a lack of blood supply in that region. Many reports exist describing successful laparoscopic ureteral reimplantations; hence, in the present case, we too opted not to perform open surgery. 17 The laparoscopy should be assisted by simultaneous cystoscopy, which facilitates the procedure tremendously. If the distance to be bypassed is less than 15 cm, the psoas hitch procedure is the optimal choice, when longer, then one should consider the Boari flap ureteroneocystostomy. 6 The extravesical uterus fixation is feasible via the laparoscopic approach, which is why we chose for a modification of Hertle's procedure. In the present case, we avoided tunneling because we had reasonable concerns that the intramural ureter could induce significant resistance for the functionally impaired dilated kidney, which would lead to renewed pressure increase. In other cases, however, it is recommended not to dispense with tunneling to protect for potential reflux. This can also be done laparoscopically because of the extravesical location.

A conservative hormone therapy without surgical cleanup of endometriomas is only justified when the urogenital endometriosis still displays an adequate renal function and is continuously monitored. 18

## Summary

In severe and recurrent cases of endometriosis with affected ureter and impaired renal function, a surgical intervention is required. However, when this treatment does not provide permanent success, there is a risk for loss of renal function, and pain and degree of discomfort are high, a laparoscopic ureteral reimplantation can be a promising therapeutic approach.

## References

[1] Giudice L C Clinical practice. Endometriosis N Engl J Med 2010 362 25 2389 2398 20573927 10.1056/NEJMcp1000274 PMC3108065

[2] Jubanyik K J Comite F Extrapelvic endometriosis Obstet Gynecol Clin North Am 1997 24 02 411 440 9163774 10.1016/s0889-8545(05)70311-9

[3] Wang P Wang X P Li Y Y Hydronephrosis due to ureteral endometriosis in women of reproductive age Int J Clin Exp Med 2015 8 01 1059 1065 25785093 PMC4358548

[4] (DGGG) DGfGuG.AWMF online. 015/045-S2k-Leitlinie: Diagnostik und Therapie der Endometriose2013;Stand: 08/2013

[5] Maccagnano C Pellucchi F Rocchini L Ureteral endometriosis: proposal for a diagnostic and therapeutic algorithm with a review of the literature Urol Int 2013 91 01 1 9 23689345 10.1159/000345140

[6] Hertle L Becht E Jacobi G H Riedmiller H Hohenfellner R Universelle Ureterozystoneostomie nach der Psoas-Hitch-Technik Indikation - Operationstechnik Aktuelle Urol 1983 14 04 167 174

[7] Dreikorn K RL Nierentransplantation in Hohenfellner R, Zingg EJ Urologie in Klinik und Praxis Thieme Verlag Stuttgart 1983

[8] Saavalainen L Heikinheimo O Tiitinen A Härkki P Deep infiltrating endometriosis affecting the urinary tract-surgical treatment and fertility outcomes in 2004-2013 Gynecol Surg 2016 13 04 435 444 28003801 10.1007/s10397-016-0958-0 PMC5133280

[9] Knabben L Imboden S Fellmann B Nirgianakis K Kuhn A Mueller M D Urinary tract endometriosis in patients with deep infiltrating endometriosis: prevalence, symptoms, management, and proposal for a new clinical classification Fertil Steril 2015 103 01 147 152 25439849 10.1016/j.fertnstert.2014.09.028

[10] Abbott J Hawe J Hunter D Holmes M Finn P Garry R Laparoscopic excision of endometriosis: a randomized, placebo-controlled trial Fertil Steril 2004 82 04 878 884 15482763 10.1016/j.fertnstert.2004.03.046

[11] Garry R The effectiveness of laparoscopic excision of endometriosis Curr Opin Obstet Gynecol 2004 16 04 299 303 15232483 10.1097/01.gco.0000136496.95075.79

[12] Deguara C S Pepas L Davis C Does minimally invasive surgery for endometriosis improve pelvic symptoms and quality of life? Curr Opin Obstet Gynecol 2012 24 04 241 244 22729092 10.1097/GCO.0b013e328355626f

[13] Johnson N P Hummelshoj L; World Endometriosis Society Montpellier Consortium.Consensus on current management of endometriosis Hum Reprod 2013 28 06 1552 1568 23528916 10.1093/humrep/det050

[14] Urdl W Der derzeitige Stand der konservativen Therapie der Endometriose J Reproduktionsmed Endokrinol 2006 3 01 24 30

[15] Donnez J Nisolle M Squifflet J Ureteral endometriosis: a complication of rectovaginal endometriotic (adenomyotic) nodules Fertil Steril 2002 77 01 32 37 11779587 10.1016/s0015-0282(01)02921-1

[16] Nezhat C Paka C Gomaa M Schipper E Silent loss of kidney seconary to ureteral endometriosis JSLS 2012 16 03 451 455 23318072 10.4293/108680812X13462882736213 PMC3535807

[17] Bourdel N Cognet S Canis M Laparoscopic ureteroneocystostomy: be prepared! J Minim Invasive Gynecol 2015 22 05 827 833 25850073 10.1016/j.jmig.2015.03.019

[18] Efe E Bakacak M Serin S Kolus E Ercan O Resim S Hormonal treatment for severe hydronephrosis caused by bladder endometriosis Case Rep Urol 2014 2014 14 891295 25506035 10.1155/2014/891295 PMC4251884

